# Actual trends in palliative oncologic surgery

**DOI:** 10.1186/1471-2318-11-S1-A40

**Published:** 2011-08-24

**Authors:** G Patrizi, G Miscusi, F Pelle, G Di Rocco, L Venturini, D Giannotti, F Frezzotti, R Giordano, A Redler

**Affiliations:** 1 Dipartimento di Scienze Chirurgiche, Policlinico Umberto I, Università di Roma“La Sapienza”, Rome, Italy

## Background

Geriatric oncologic surgery is frequently based on palliative treatments. Few studies focus on the advantages and pitfalls of surgical versus endoscopic palliation in oncological surgery. The reviews published on this topic and a statistical non-specific general profile of our Institution’s experience may depict a realistic and current general trend.

## Materials and methods

Data from the literature were revised, focusing our attention on published reviews on surgical and endoscopic palliation for oesophagus, colon-rectum and pancreatic-biliary cancers. We also obtained the data from the registries of our hospital on these very diagnoses. Finally, we requested the number of metallic stents sold in Italy and in Europe to one of the main manufacturers, assuming that almost all of them are for oncological palliation.

## Results

Even though most of the published reviews conclude favorably for a surgical palliation, the general trend appears to be more and more oriented towards the endoscopic palliation with stents. Little was written on the potential role of laparoscopy for surgical palliation.

## Conclusions

There is a growing request for endoscopic treatments, strengthened by a prioritary scientific interest and technological development in this field, with growing responsibility for endoscopists and interventional radiologists.

